
JOURNAL INFORMATION
==============================
NLM Title Abbreviation: CPT Pharmacometrics Syst Pharmacol
Journal ID: CPT Pharmacometrics Syst Pharmacol
NLM Journal ID: 101580011
Journal ID: PSP4

CPT: Pharmacometrics & Systems Pharmacology

EISSN: 2163-8306
Publisher: Wiley

ARTICLE INFORMATION
==============================
PMCID: PMC11474762
DOI: 10.1002/psp4.12822
Article ID: PSP412822
Article version: 1
Subjects: Issue Information, Issue Information

Issue Information

Electronic publication date: 2023 Jul 14
Publication date: 2023 Jul
Volume: 12
Issue: 7
First page: 879
Last page: 882
Copyright: © 2023 The Authors. CPT: Pharmacometrics & Systems Pharmacology published by Wiley Periodicals LLC on behalf of American Society for Clinical Pharmacology and Therapeutics.
License: This is an open access article under the terms of the http://creativecommons.org/licenses/by/4.0/ License, which permits use, distribution and reproduction in any medium, provided the original work is properly cited.
License URL: https://creativecommons.org/licenses/by/4.0/

Figure count: 0
Table count: 0
Page count: 3

source-schema-version-number: 2.0
cover-date: July 2023
details-of-publishers-convertor: Converter:WILEY_ML3GV2_TO_JATSPMC version:6.4.9 mode:remove_FC converted:15.10.2024

==============================

An Official Journal of ASCPT and ISoP

### CPT: Pharmacometrics & Systems Pharmacology

Open Access

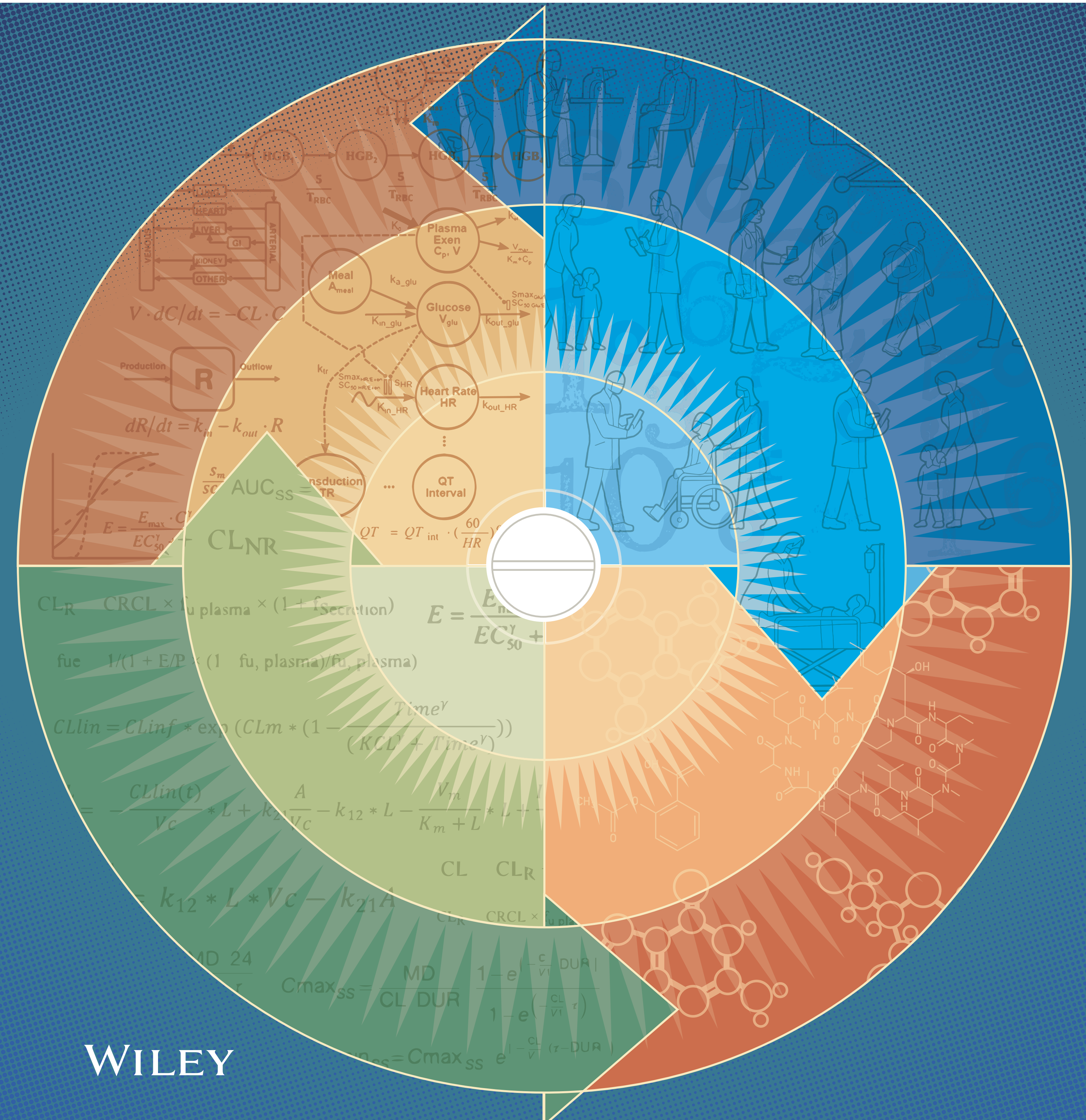

WILEY

### CPT: Pharmacometrics & Systems Pharmacology

WILEY

### Editor-in-Chief

France Mentré, MD, PhD, *School of Medicine of University Paris Diderot, Paris, France*

### Deputy Editor-in-Chief

Karen Rowland Yeo, PhD, *Certara UK Ltd., Sheffield, UK*

### Associate Editors

Jonathan French, ScD, *Metrum Research Group, Tariffville, CT, USA*
Stefanie Hennig, MSc, PhD, *Certara Strategic Consulting, Brisbane, Australia*
Sriram Krishnaswami, PhD, *Pfizer, Groton, CT, USA*
Vikram Sinha, PhD, *Takeda Pharmaceuticals International Co., Cambridge, MA, USA*
Ping Zhao, PhD, *Bill & Melinda Gates Foundation, Seattle, WA, USA*
Eric Sobie, PhD, *Metrum Research Group, Tariffville, CT, USA*

### Editors-in-Training

Rajat Desikan, PhD, *GSK, UK, Middlesex, UK*
Priya Jayachandran, PharmD, *Regeneron Pharmaceuticals, Inc., San Francisco, CA, USA*

### American Society for Clinical Pharmacology and Therapeutics

***Chief Executive Officer***
Abigail W. Gorman, *Alexandria, VA*
***Chief Operating Officer***
Elise S. Laffman-Johnson, *Alexandria, VA*
***Managing Editor***
Alethea B. Gerding, *Alexandria, VA*
***Deputy Managing Editor***
Alaina Webster, *Alexandria, VA*
***Senior Editorial Coordinator***
Aimee Greene, *Alexandria, VA*

### Editorial Board

Antoinette Ajavon-Hartmann, PhD, *Bristol Myers Squibb, Lawrence Township, NJ, USA*
Vivaswath S. Ayyar, PhD, *Janssen Research & Development, Spring House, PA*
Joseph P Balthasar, PhD, *University of Buffalo Buffalo, NY, USA*
Robert Bies, PharmD, PhD, *State University of New York at Buffalo, Buffalo, NY, USA*
Marc Birtwistle, PhD, *Clemson University Clemson, SC, USA*
Marylore Chenel, PharmD, PhD, *Pharmetheus Uppsala, Sweden*
Emmanuel Chigutsa, PhD, *Eli Lilly and Company, Indianapolis, IN, USA*
Teresa Dalla Costa, PhD, *Federal University of Rio Grande do Sul - UFRGS Porto Alegre, Brazil*
Paolo Denti, PhD, *University of Cape Town Cape Town, South Africa*
James M. Gallo, PharmD, PhD, *University at Buffalo, Buffalo, NY, USA*
Wei Gao, PhD, *EMD Serono, Billerica, MA, USA*
Ekaterina Gibiansky, PhD, *QuantPharm LLC North Potomac, MD, USA*
Manish Gupta, PhD, FCP, *GenMab; Princeton, NJ, USA*
Bart Hendriks, PhD, *Novartis Institutes for BioMedical Research Cambridge, MA, USA*
Nick Holford, MBChB, *The University of Auckland Auckland, New Zealand*
Brett A. Howell, PhD, *DILIsym Services Division, Simulations Plus, Inc., Research Triangle Park, NC, USA*
Manuel Ibarra, PhD, *Universidad de la República, Montevideo, Uruguay*
Siv Jönsson, PhD, *Uppsala University Sweden, Uppsala, Sweden*
Kristin Karlsson, PhD, *Uppsala University Uppsala, Sweden*
Helen Kastrissios, PhD, *QuanTx Consulting, Brisbane, Australia*
Ron Keizer, PhD, *InsightRX San Francisco, CA, USA*
Steven E. Kern, PhD, *Global Health Labs, Bellevue, WA, USA*
Andrzej Kierzek, PhD, *Certara QSP; Sheffield, United Kingdom*
Qi Liu, PhD, *US Food and Drug Administration, Silver Spring, MD*
Rajanikanth (Raj) Madabushi, PhD, *US Food and Drug Administration, Silver Spring, MD*
Efthymios Manolis, MSc, *European Medicines Agency London, United Kingdom*
Peter Milligan, PhD, *Pharmetheus, Uppsala, Sweden*
Jackson K. Mukonzo, PhD, *Makerere University, Kampala Uganda*
Joakim Nyberg, PhD, *Pharmetheus, Uppsala, Sweden*
Kyungsoo Park, MD, PhD, *Yonsei University College of Medicine Seoul, Republic of Korea*
Elsje Pienaar, PhD, *Weldon School of Biomedical Engineering, Purdue University, West Lafayette, IN, USA*
Jose Pinheiro, PhD, *Johnson and Johnson Pharmaceutical Research Raritan, NJ, USA*
Benjamin Ribba, PhD, *F. Hoffman-La Roche, Basel, Switzerland*
Radojka Savic, PhD, *University of California, San Francisco, San Francisco, CA, USA*
Daniel Scholes Rosenbloom, PhD, *Merck & Co., Rahway, NJ, USA*
Joe Standing, PhD, *University College London London, United Kingdom*
Natasha Strydom, PhD, *Amgen, Inc., San Francisco, CA, USA*
Yuichi Sugiyama, PhD, *Riken Innovation Center Sugiyama Laboratory Yokohama, Japan*
Joel Tarning, PhD, *Mahidol-Oxford Tropical Medicine Research Unit Bangkok, Thailand*
Michael Tortorici, PharmD, PhD, *Aro Biotherapeutics, Philadelphia, PA, USA*
Paolo Vicini, PhD, MBA, *Confo Therapeutics, Ghent, Belgium*
Camille Vong, PhD, *Novartis AG Pharma, Basel, Switzerland*
Ping Zhang, PhD, *The Ohio State University, Columbus, OH, USA*
Xinyuan (Susie) Zhang, PhD, *Daiichi Sankyo, Inc. Basking Ridge, NJ, USA*

### Overview

*CPT: Pharmacometrics & Systems Pharmacology* (PSP) is an official open access journal of the American Society for Clinical Pharmacology and Therapeutics (ASCPT) and International Society of Pharmacometrics (ISoP).

*CPT: Pharmacometrics & Systems Pharmacology* is a cross-disciplinary journal devoted to publishing advances in quantitative (e.g., modeling and simulation) methods as applied in pharmacology, physiology and therapeutics in humans. The journal welcomes original research articles, reviews and tutorials that bridge the following areas: pharmacometrics, modeling and simulation as applied to the design and evaluation of clinical trials, systems pharmacology modeling, particularly with a mechanistic link to human (patho)physiology, disease modeling, “population” or mixed-effects pharmacokinetics and pharmacodynamics (PKPD) modeling, modeling and simulation to support translational research, physiologically-based pharmacokinetics (PBPK), model-based meta-analyses of clinical trials, mechanism-based pharmacokinetic-pharmacodynamic modeling, computational pharmacology, bioinformatics, comparative efficacy, effectiveness and cost-effectiveness. Systems pharmacology may involve the application of systems biology approaches to study drug activities, targets and effects. The discipline is often defined with reference to engineering and pharmacological principles as the quantitative analysis of the dynamic interactions between drugs and a biologic system that aims to understand the behavior of the system as a whole. The common focus will be on quantitative methods that improve our understanding of pharmacology and therapeutics in humans.

**Abstracting and Indexing**
- Embase (Elsevier)
- SCOPUS (Elsevier)
- MEDLINE/PubMed indexing

**Society Information**
The American Society for Clinical Pharmacology and Therapeutics (ASCPT) was founded in 1900, and consists of over 2,200 professionals whose primary interest is to advance the science and practice of clinical pharmacology and translational medicine for the therapeutic benefit of patients and society. ASCPT is the largest scientific and professional organization serving the disciplines of Clinical Pharmacology and Translational Medicine.

**All articles published in this journal are freely available to read, download, and share. Articles are published under the terms of the Creative Commons Attribution Non-Commercial License which permits use, distribution, and reproduction in any medium, provided the original work is properly cited and is not used for commercial purposes.**

**Disclaimer**
The Publisher, the American Society for Clinical Pharmacology and Therapeutics, the International Society of Pharmacometrics, and Editors cannot be held responsible for errors or any consequences arising from the use of information contained in this journal; the views and opinions expressed do not necessarily reflect those of the Publisher, the American Society for Clinical Pharmacology and Therapeutics, the International Society of Pharmacometrics or Editors, neither does the publication of advertisements constitute any endorsement by the Publisher, the American Society for Clinical Pharmacology and Therapeutics, the International Society of Pharmacometrics or Editors of the products advertised.

**Publisher**
*CPT: Pharmacometrics & Systems Pharmacology* is published by Wiley Periodicals, LLC, 101 Station Landing, Suite 300, Medford, MA 02155; Telephone: 781-388-8200; Fax: 781-388-8210.

CONTENTS

CPT: Pharmacometrics & Systems Pharmacology

### PERSPECTIVE

### PERSPECTIVE

**883** Requirements, expectations, challenges and opportunities associated with training the next generation of pharmacometricians
**Stephan Schmidt, Valvanera Vozmediano, Rodrigo Cristofoletti, Sarah Kim, Zhoumeng Lin, Natalia de Moraes, Francine Azeredo, Brian Cicali, Hans Leuenberger, Joshua D. Brown, Jin Y. Jin, Cynthia J. Musante, Stacey Tannenbaum and Yaning Wang**

### TUTORIAL

### TUTORIAL

**889** QSP Designer: Quantitative systems pharmacology modeling with modular biological process map notation and multiple language code generation
**Richard J. Matthews, David Hollinshead, Daniel Morrison, Piet H. van der Graaf and Andrzej M. Kierzek**

### RESEARCH

### ARTICLES

**904** Evaluation of model-based bioequivalence approach for single sample pharmacokinetic studies
**Coralie Tardivon, Florence Loingeville, Mark Donnelly, Kairui Feng, Wanjie Sun, Guoying Sun, Stella Grosser, Liang Zhao, Lanyan Fang, France Mentré and Julie Bertrand**

**916** A decision support system based on artificial intelligence and systems biology for the simulation of pancreatic cancer patient status
**Valentin Junet, Pedro Matos-Filipe, Juan Manuel García-Illarramendi, Esther Ramírez, Baldo Oliva, Judith Farrés, Xavier Daura, José Manuel Mas and Rafael Morales**

**929** Model-based approach to identify predictors of paclitaxel-induced myelosuppression in "real-world" administration
**Ahmed M. Salem, Erik Dvergsten, Sanja Karovic, Michael L. Maitland and Mathangi Gopalakrishnan**

**941** Development of a PBPK model to quantitatively understand absorption and disposition mechanism and support future clinical trials for PB-201
**Miao Zhang, Zihan Lei, Ziheng Yu, Xueting Yao, Haiyan Li, Min Xu and Dongyang Liu**

**953** Synthetic Model Combination: A new machine-learning method for pharmacometric model ensembling
**Alexander Chan, Richard Peck, Megan Gibbs and Mihaela van der Schaar**

**963** Population PK/PD model of tacrolimus for exploring the relationship between accumulated exposure and quantitative scores in myasthenia gravis patients
**Di Chen, Qingyu Yao, Wenjun Chen, Jian Yin, Shifang Hou, Xiaoxin Tian, Ming Zhao, Hua Zhang, Liping Yang, Tianyan Zhou and Pengfei Jin**

**977** Model-informed pediatric dose selection of marzeptacog alfa (activated): An exposure matching strategy
**Alan Faraj, Rob C. van Wijk, Linda Neuman, Shraddha Desai, Grant E. Blouse, Tom Knudsen and Ulrika S. H. Simonsson**

**988** A population pharmacokinetic and pharmacokinetic-pharmacodynamic analysis of vupanorsen from phase I and phase II studies
**Jae Eun Ahn, Steven G. Terra and Jing Liu**

**1001** Physiologically based pharmacokinetic modeling of levetiracetam to predict the exposure in hepatic and renal impairment and elderly populations
**Chaozhuang Shen, Wenxin Shao, Wenhui Wang, Hua Sun, Xiaohu Wang, Kuo Geng, Xingwen Wang and Haitang Xie**

**1016** Disease progression joint model predicts time to type 1 diabetes onset: Optimizing future type 1 diabetes prevention studies
**Juan Francisco Morales, Rhoda Muse, Jagdeep T. Podichetty, Jackson Burton, Sarah David, Patrick Lang, Stephan Schmidt, Klaus Romero, Inish O'Doherty, Frank Martin, Martha Campbell-Thompson, Michael J. Haller, Mark A. Atkinson and Sarah Kim**

**1029** Modeling Alzheimer's disease progression utilizing clinical trial and ADNI data to predict longitudinal trajectory of CDR-SB
**Samira Jamalian, Michael Dolton, Pascal Chanu, Vidya Ramakrishnan, Yesenia Franco, Kristin Wildsmith, Paul Manser, Edmond Teng, Jin Y. Jin, Angelica Quartino and Joy C. Hsu for the Alzheimer's Disease Neuroimaging Initiative**
